# Effectiveness of a Multifactorial Intervention in the First 1000 Days of Life to Prevent Obesity and Overweight in Childhood: Study Protocol

**DOI:** 10.3390/ijerph17072239

**Published:** 2020-03-26

**Authors:** Mercedes Díaz-Rodríguez, Celia Pérez-Muñoz, José Manuel Lendínez-de la Cruz, Martina Fernández-Gutiérrez, Pilar Bas-Sarmiento, Bernardo C. Ferriz-Mas

**Affiliations:** 1 Department of Nursing and Physiotherapy, University of Cádiz, 11009 Andalusia, Spain; mercedes.diaz@uca.es (M.D.-R.); martina.fernandez@uca.es (M.F.-G.); pilar.bas@uca.es (P.B.-S.); 2 Ribera del Muelle Health Centre, Clinic Management Unit (CMU) Puerto Real, Cádiz, Andalusian Health System, 11510 Andalusia, Spain; jlendinezd@hotmail.com; 3 Río San Pedro Health Centre, Clinic Management Unit (CMU) Puerto Real, Cádiz, Andalusian Health System, 11519 Andalusia, Spain

**Keywords:** obesity, childhood, early programming, prevention, primary care

## Abstract

(1) *Background*: Obesity is a global health problem, and its prevention must be a priority goal of public health, especially considering the seriousness of the problem among children. It is known that fetal and early postnatal environments may favor the appearance of obesity in later life. In recent years, the impact of the programs to prevent obesity in childhood has been scarce. The aim of this research is to evaluate the effectiveness of an intervention based on the concept of early programming. (2) *Methods*: Non-randomized controlled trial design. Inclusion criteria are: two-year-old infants whose gestational period begins in the 14 months following the start of the intervention, and whose mothers have made the complete follow-up of their pregnancy in the same clinical unit of the study. The intervention will be developed over all the known factors that affect early programming, during pregnancy up to 2 years of life. Data will be collected through a data collection sheet by the paediatricians. A unibivariate and multivariate analysis of the data will be carried out. (3) *Ethics and dissemination*: The trial does not involve any risk to participants and their offspring. Signed informed consent is obtained from all participants. Ethical approval has been obtained. (4) *Results*: It is expected that this study will provide evidence on the importance of the prevention of obesity from the critical period of the first 1000 days of life, being able to establish this as a standard intervention in primary care.

## 1. Introduction

Obesity is a global public health problem, and it has been declared the epidemic of the 21st century by the World Health Assembly [1] because of its high prevalence and its association with other chronic diseases [2,3]. The development of preventive strategies must be a priority goal of public health in order to decrease the social and economic burden of obesity [4].

The situation is even more concerning among children, due to its high incidence in developed countries, the appearance of excess weight at younger ages [5,6] and the fast increase in the prevalence of childhood obesity in emerging countries [7].

Childhood obesity is recognized as a disease by the World Health Organization. It is also associated with other pathologies and can lead to psychological damage [8]. The high prevalence of obesity in childhood and adolescence becomes a problem for the health system, since a large proportion of overweight children will be obese in their adulthood [9].

In addition, inequalities in obesity have risen, with greater increases in the prevalence of obesity in the most disadvantaged social groups [10], so that obesity is a marker of social inequalities in health [11].

In Spain, the latest data in school children [12] show a high prevalence of excess weight, with a significant reduction in overweight, but not in obesity [13]. In order to fight against diseases whose causes are related to exposure to social and environmental factors, such as obesity, preventive measures provide the best results. In recent years, programs to prevent childhood obesity have been developed [14,15,16]. However, the effects of these programs on the child population have been scarced in scope [12]. In this sense, in order to confront these results, some authors propose a change in the approach of strategies to prevent childhood obesity [17,18].

Recent knowledge about early programming offers the opportunity to develop new prevention strategies. The concept of early programming has been incorporated into the prevention recommendations for childhood obesity [19,20]. These new strategies should be focused on the maximum-plasticity period of early programming, before and during pregnancy and in the first 2–3 years of postnatal life. This period is critical both for development and for the prevention of childhood obesity.

A systematic review [21] of interventions aimed at preventing childhood obesity, developed in pregnancy and the first 2 years of life, only found effectiveness in interventions based on the protein content of the formulas or in behavioral interventions focused on parents about nutrition and responsible feeding.

Another systematic review of interventions during the first 1000 days found a total of 26 interventions [22]. Two were only developed during pregnancy, and were not effective. Six began during pregnancy and continued in the postnatal stage. Of these, those involved in only one aspect (breastfeeding promotion, nutritional supplements) were not effective. Two interventions, based on home visits and group sessions focused on diet, infant feeding practices and physical activity, proved effective on BMI at 12 or 24 months. Therefore, it is desirable that interventions take place over the entire 1000-day period and encompass as many aspects as possible related to the risk of childhood obesity.

The fact that an early exposure to environmental factors could influence the health of the offspring in future life has been recognized from the first half of the 20th century, with the epidemiological studies of Kermack [23]. Later, Barker formulated the hypothesis of the fetal origin of adult diseases, which relates the incidence of certain adult diseases to an environment of malnutrition within the uterus [24]. Early programming is considered an adaptive response to the environment, based on the information received during fetal life and the first years of postnatal life [25].

Several studies in humans and animal models propose different early programming mechanisms [26,27,28]. In recent years, there has been great interest in the role of epigenetics. This is defined as the set of non-genetic factors that induce heritable changes in the expression of genes without altering the sequence of the genome [29]. This susceptibility to early programming is limited to a critical period of development, especially in fetal life and the first 2–3 years of postnatal life [30,31].

Several early programming factors (EPF) have been associated with childhood and adult obesity. They can be classified into pre- and post-natal factors:

Prenatal: maternal overweight or obesity [32,33,34], excessive weight gain during pregnancy [34,35,36], gestational diabetes [37,38], maternal malnutrition [39,40,41] and maternal smoking [42,43].

Postnatal: short breastfeeding [44], rapid weight gain in the first year of life [45], excess protein intake [5,46], vitamin D deficiency [47,48], and caesarean section (where there is evidence for and against) [49,50,51].

Other factors, related to lifestyle and family eating behaviors, have also been linked to an increased risk of obesity in childhood: appetite control [52,53], sleeping habits [54], quality of maternal-child relationship [55] and other modifiable factors like screen time, physical activity [56], complementary feeding [57], etc. 

The accumulation of these early programming factors in the same individual is associated with a progressive risk of obesity at 6 years old [58,59] or at 7–10 years old [60], regardless of what these factors are. Although all factors that are associated with childhood obesity cannot be controlled, the decrease in the number of factors that accumulate in an individual could reduce the obesity risk.

Accordingly, prevention strategies are more effective the earlier they have been developed in the life course. Therefore, we have developed strategies that are implemented from the prenatal stage until 2 years old. The concept of early programming must be used alongside the prevention opportunity, with a positive message that obesity prevention is possible by decreasing the number of programming factors. In this project, we want to carry out a realistic intervention, developed entirely in primary care by the usual health team. It must be acceptable to primary care professionals and supported and endowed with resources by the health administration. It also must be integrated into the routine practice of the Andalusian Children and Adolescents Health Program (A-CAHP) and Care Process of Pregnancy, Childbirth and Puerperium (CPPCP) controls, taking advantage of the high number of visits planned in these programs, so that it can, if proves to be effective, generalize to routine activity in primary care. 

The project’s global hypothesis is that an intervention in the first 1000 days of life on the early programming factors related to childhood obesity will achieve more adequate early programming and will decrease the prevalence of childhood obesity. The risk of developing obesity in childhood will be lower when the number of factors accumulated in the individual in this critical first phase is decreased. This improvement in early metabolic programming will be reflected in a lower percent of body fat mass at 2 years of age, compared to the control group.

The aim of the project is to evaluate the effectiveness of a combined multifactorial educational and care intervention for preventing obesity and overweight in childhood, based on the concept of early programming, and developed during pregnancy and the first 2 years of life (the first 1000 days) by a multidisciplinary group in primary care. The specific objectives are:

To analyse the body fat mass at 2 years of age in a control group of children who reach this age between November 2017 and June 2019, belonging to two pediatric quotas of the Basic Health Zone (BHZ) of Puerto Real.

To analyse the body fat mass at 2 years of age in the intervention group of children, whose pregnancies begin between January and December 2018, belonging to four pediatric quotas of the BHZ of Puerto Real. 

To compare the body fat mass at 2 years of age between the control and intervention groups.

## 2. Materials and Methods 

### 2.1. Study Design

This study is a quasi-experimental design with two groups (control and experimental group), in which the results on body fat in 2-year-old children, obtained after a combined educational and care intervention, are compared with those obtained after the usual intervention developed in our Clinical Management Unit (CMU). 

Randomization could not be carried out for ethical reasons; once the intervention has begun, we should not deprive a part of the population of it. Similarly, as it is a small town, there could be contamination between groups (transfer of information). Therefore, a historical control group of the same population consisting of children who turn 2 years old during 2018 will be used.

### 2.2. Study Population

The target population is two-year-old infants. The BHZ of Puerto Real (Cádiz) is constituted by two CMUs: Puerto Real, with four pediatric quotas and Casines, with two. The project will be developed in the CMU Puerto Real. There are no significant sociocultural differences between the different quotas. Since the number of births in the BHZ during the previous year was 320, it is expected that, on average, 50 newborns will be added annually to each quota. 

### 2.3. Masking 

Due to the characteristics of the study, it is not possible to blind it to the participants or to the researchers who will carry out the intervention. However, it will be masked for researchers, who will perform the evaluation of the effectiveness and analysis of the data. To do this, they will not know the coding of the randomization variable, which will be guarded. This way, it will not be possible for them to know which of the groups received the intervention until the analysis is completed.

### 2.4. Recruitment

A single midwife will take charge of maternal education in the CMU Puerto Real, which includes the Ribera del Muelle Health Centre where there are three paediatricians, and the Rio San Pedro Health Centre where there is one. Therefore, all pregnant women will receive exactly the same information about the intervention. After birth, the parents will decide in which of the six paediatricians of the BHZ of Puerto Real they will register their children, and we cannot interfere in that decision.

The study population of the control group will be constituted by two-year-old children of two quotas of the Basic Health Zone of Puerto Real. The paediatricians have employment stability for at least the expected duration of the study and have agreed to participate. The inclusion in the study and the data collection will be carried out in the health controls at 2 years of age at the same time as the intervention in pregnant women begins, so that neither mothers during pregnancy nor infants after delivery will have been influenced by the intervention. Once the intervention ends, their results will be analyzed from the data obtained from the mother–child pairs.

Following the above criteria, all newborns assigned to the four pediatricians of the unit that meet the inclusion criteria will be included in the intervention group. Two paediatricians from the same unit will also participate in the data collection for the control group. Including a greater number of pediatricians is necessary to obtain the calculated sample of the intervention group, due to the decrease in the birth rate observed in recent years. Inclusion in the study will be done at the first pediatric visit of the newborn.

The inclusion criteria are: two-year-old infants whose pregnancy begins in the 14 months following the start of the intervention, and whose mothers have made the complete follow-up of their pregnancy in the Care Process of Pregnancy Childbirth and Puerperium and begun the monitoring of child health controls of the Andalusian Children and Adolescents Health Program in our unit. The exclusion criteria are: infants whose parents do not authorize their participation in the study in writing.

### 2.5. Sample Size Estimation

An intentional sampling has been performed on a population of 320 births over a period of time of 1 year (2017) in the geographical situation of Puerto Real. The source of information was historical data collected from primary care management of the Bahía de Cádiz-La Janda District. It is estimated that the control group will be formed of 100 sample units and the experimental group of 150 sample units. For this configuration, assuming the randomness of the sample collected, the precision errors will be approximately 9% and 7% respectively (from the experimental group and the control group). These error levels were calculated by establishing a significance level of 95% (α = 0.05) and assuming a loss rate of 15%.

We estimate that this number of cases will guarantee the level of confidence and power necessary for statistical analysis.

### 2.6. Retention

The control group does not need incentives to continue in the study since, once their participation is accepted, the data of the mother–child pairs are taken and do not require any other action. The participants of the intervention group, once their participation in the first month’s visit has been accepted, only have to go to the usual health checks until 2 years. At these ages, parents are very receptive to participate in all initiatives that can improve the health of their children, especially if they are developed by the usual professionals. We consider that they will remain in the study without difficulty.

### 2.7. Intervention 

The intervention is being carried out during pregnancy and the following 2 years after birth. The intervention period during pregnancy began, for each individual patient, when they went to their first visit related to the Care Process of Pregnancy, Childbirth and Puerperium Program during the period of time between January 2018 and February 2019. This first visit is carried out by the midwife in charge (Clinical Managing Unit) and is integrated within the standard medical visits and workshops, although reinforced by a specific workshop, purely based on the intervention scheme, at the beginning of the patient’s pregnancy. 

Three workshops take place during this period. They happen once a month for a total of two hours at a time. In each workshop, the patients are handed a fully informed leaflet in which they can learn about the information given and write personal notes at their discretion. 

Workshop number one, specifically created for the intervention period, takes place before the 12th week of pregnancy is reached. To begin the workshop, a 15- to 20-min slideshow is shown to explain the “early programming” concept and its close relation to childhood obesity. Immediately after this, the attendees will be fully informed about the importance of physical exercise, quitting toxic habits and creating a healthy eating plan for the entire family. 

At the second workshop, which has to be completed between the 16th and 18th weeks of pregnancy, the information provided is related to the specific healthcare and healthy habits that a pregnant woman should embrace, considering the changes she will experience.

Finally, on the third workshop, carried out between the 30th and 32nd weeks of pregnancy, the attendees will learn about the special characteristics of care related to newborns. To begin, a 20-min slideshow, in which the postnatal programming and its different factors are explained in detail, will be shown. After that, exclusive breast feeding will be explained and promoted as the preferable option. The child and adolescent health scheme planed by the Andalusian health authorities will also be introduced during this session. 

The first visit within the pregnancy, birth and puerperium scheme is carried out between the 4th and 7th week of pregnancy. It is a 30-min visit in which the pregnant woman will receive an informative leaflet with general recommendations about a healthy pregnancy, where she will be introduced to the concept of early programming and its close relation to childhood obesity. Those pregnant women considered obese, who admit smoking, or who are believed to be unhealthy dieters, will be encouraged to take on the first workshop. 

Subsequent visits will monitor the risk factors, including whether new factors arise or persist, in which case workshop number one will be offered again or as a first time informative session. Early programming and childhood obesity will be the main and consistent ideas to be reinforced on each visit. The basic recommendations and lifestyle habits proposed to each pregnant woman will be related to these core concepts. 

Both workshops two and three will be offered to all pregnant women in the program. Those in the 14th–16th week of pregnancy will be encouraged to attend workshop number two, and those in the 30th-32nd week will be offered workshop number three.

Recommendations during the first year:

1. Promotion of breastfeeding (exclusively up to 6 months).
  - Participation in local breastfeeding support groups.
  - Avoidance of active/passive maternal smoking.
  - Information on the advantages of breastfeeding for the prevention of childhood obesity.
2. Recommendations for artificial lactation:
  - Formulas with lower protein content are recommended up to 12 months, with a strong recommendation for children of overweight or obese mothers, gestational diabetes, and macrosomic newborns, if breastfeeding is not possible.
  - Introduce complementary feeding between 4 and 6 months of life.
  - Bottle feed should be slow, at least 15-20 min.
3. Supplementary feeding tips:
  - Reduce the protein content of animal origin between 6-12 months: 15 g/day of meat at 6 months and increase up to 40 g/day at the end of the first year.
  - Tips to facilitate the introduction of fruits and vegetables to favor a more varied diet in the preschool stage
  - Avoid the introduction of sweet flavors: honey, infusions, juices, etc.
4. Tips for developing self-regulation of appetite/satiety.
  - Identify signs of appetite/satiety in the infant.
  - Do not force eating.
  - Do not calm crying or night awakenings systematically with food.
5. Vitamin D supplement (400 IU/day) during the first year.
6. Tips for developing a secure attachment.
7. Tips to develop adequate sleep habits.
8. Recommendation of active play after 6 months.
9. Avoid using screens, especially for sleeping and during meals.

Recommendations during the second year:

1. Recommend growth formulas because of their lower protein content, compared to cows’ milk.
2. Limit milk intake to about 500 mL per day.
3. Restrict the intake of sweet flavors.
4. Restrict the bottle as soon as possible.
5. Maintain the recommendations in items 7–9 of the first year.

Most of the recommendations proposed in the intervention are not novel themselves, except for the recommendation to feed formulas with lower protein content to infants who are not breastfeeding. 

The strength of the intervention is given by:

1. The coordinated action of all professionals who attend pregnancy and the first 2 years of postnatal life, offering recommendations about healthy lifestyles, guidelines on suitable nutrition and proper acquisition of habits. Using the concept of early programming as an opportunity for primary prevention of obesity and other chronic diseases.
2. The use of the concept of early programming as the central axis of the intervention, associated with an opportunity to carry out a primary prevention of obesity and other chronic diseases.
3. Explaining to the families, in this stage of great receptivity to the recommendations on health care, that the care guidelines for your children can have permanent and long-term consequences on their health.

### 2.8. Measures and Outcomes

#### 2.8.1. Independent Variables

1. Weight gain during pregnancy. The difference in maternal weight between the first visit (weeks 4–7) and the last one (week 38) is calculated. If the weight on the last visit is not registered, it is calculated with the final pregnancy weight recorded in the hospital discharge report. It will be classified as adequate or excessive according to the accepted values of weight increase by the pre-gestational body mass index (BMI) of the Institute of Medicine (IOM) and National Research Council [61].
2. Mother’s body mass index (BMI) at the first visit (weeks 4−7). This will be evaluated as a continuous measure.
3. Maternal nutritional status. Classified as obesity, overweight, normal weight or low weight.
4. Maternal smoking during pregnancy. Active smoker, passive smoker and non-smoker. The mother is considered an active smoker if she consumes any amount of tobacco.
5. Gestational diabetes.
6. Caesarean section.
7. Newborn sex.
8. Newborn weight. Registered in hospital visit (newborn measures).
9. Duration of exclusive breastfeeding. Full months of exclusive breastfeeding are considered.
10. Weight gain in the first four months. The difference in the infant’s weight between birth weight and fourth-month-postnatal weight at the visit is calculated as grams/day. As secondary outcomes, weight gain in the first six months and in the first year are also calculated.
11. Introduction of complementary food, before or after 4 months of age.
12. Vitamin D supplementation (400 IU/day) in the first year. Full months of supplementation are considered.
13. Type of milk in second year. Classified according to the type of milk that was consumed the largest number of months: breastfeeding, growing milk or cow milk.
14. BMI at 1 year of life.

#### 2.8.2. Dependent Variables

1. BMI at 2 years of life.
2. Percentage of fat mass at 2 years of life.

### 2.9. Data Collection 

The data for the control group will be collected through a data collection sheet, specifically designed for the study, from three sources:

(1) Direct questions to the parents at the one-year and two-year visits, performed by paediatricians.
(2) The weight, height and BMI records of the Diraya program, both for pregnant women and children.
(3) Mothers and children’s medical records from the Diraya program. The form will be subsequently computerized.

The data for the intervention group will be collected in the same way, but also at the newborn and six-month visits, developed by paediatricians.

The midwives in the usual CPPCP controls will collect the gestational and delivery variables. Nurses and paediatricians in the usual controls of the A-CAHP will collect the somatometric variables of newborns up to 2 years.

After childbirth, during the puerperal visit, the midwife will register the birth data in the Diraya program as a hospital visit. If the mother does not attend the puerperal visit, it will be the paediatricians at the newborn visit who will collect the data from birth.

The weight and height of pregnant women will be measured with an ADE (GmbH & Co, Hamburg, Germany) scale, model M304641-01, with a reading range from 2 to 250 kg and a precision of 100 g, with an ADE stadiometer coupled to the scale, model MZ10023-1, with a reading range of 60–210 cm and a precision of 0.1 cm.

Weights will be measured in the first and second year of age with the child naked on a Soehnle baby scale, reading in a range of 0–20 kg with a 10 g precision. The length will be measured with the infant in supine position with a rigid Añó-Sayol stadiometer, with a reading range of 25 to 90 cm and an accuracy of 0.5 cm. The tables of weight, length and BMI values of the WHO will be used.

The skin folds will be measured with a Holtain Skinfold Caliper plicometer with an amplitude of 0–46 mm, 0.2 mm graduation and a constant pressure of 10 g/mm^2^. They will be measured in the left side of the body, in triplicate and by a single observer for each pediatric quote, according to universally recommended techniques [62,63].

### 2.10. Data Analysis

For the statistical analysis of these data, the statistical program SPSS Version 22 software (IBM Corp, Armonk, NY, USA, 2015) will be used:

Descriptive analysis. The means and standard deviations for the quantitative variables and the absolute and relative frequencies for the qualitative ones will be determined.

Bivariate analysis. The Kolgomorov–Smirnov test will be used to determine if the quantitative variables are parametric; when the sample is less than 50, the Shapiro–Wilk test will be used. For the comparison of the dependent variables with qualitative independent ones, we will use the Student’s *t*-test and ANOVA for independent means as a test of statistical significance, and the Mann–Whitney U and the Kruskall–Wallis tests for the nonparametric ones. For independent quantitative variables, the Pearson and Spearman coefficients will be used.

Multivariate analysis. The analysis of the degree dependent variables with the independent variables will be carried out using the generalized linear model. For the linear regression, we will use the Gaussian family and as a function of the identity bond and for the binary logistic regression, the binomial family and the logit link function.

To detect differences, a significance level *p* < 0.05 will be used and the 95% confidence intervals will be calculated.

### 2.11. Limitations

The main limitation of this study is non-randomness. Randomness is not possible, due to the ethical reasons described above, and because the parents have the freedom and the right to choose the paediatrician that will care for the child. However, this is compensated for by the fact that it is a homogeneous population for the control and the intervention group, which will reduce the risk of bias.

## 3. Ethics and Dissemination 

The trial does not involve any risk to participants and their offspring. Children who are not included in the study at birth are not left unprotected, but continue to receive the recommendations and reviews that are currently developed within the A-CAHP. Signed informed consent is obtained from all participants: mothers and parents or legal guardian as representatives of children, both in intervention and control groups (Supplementary A–C). The rights, privacy and integrity of the participants are guaranteed. The investigation will be carried out in accordance with the precautionary principle, to prevent and avoid risks to life and health, observing compliance with the recommendations set forth in the Declaration of Helsinki. There is authorization from the primary care management of the Bahía de Cádiz-La Janda District, and ethical approval was obtained from the Regional Committee of Ethics (Comité Coordinador de Ética de la Investigación Biomédica de Andalucía, CCEIBA) (Supplementary D). 

There is a high percentage of women who begin pregnancy with overweight or obesity. Due to the public health problem posed by obesity in the world and the lack of effectiveness demonstrated by the current interventions, it is necessary to design and implement interventions aimed at the prevention of obesity with a different perspective. This intervention represents a new approach in a key period for the development of obesity, in which few interventions have been developed in this regard. For all this, it is expected that this study will provide evidence on the importance of the prevention of obesity from the critical period of the first thousand days of life, being able to establish as a standard intervention in primary care. Results from this study will be disseminated at international congresses, in regional and national conferences and in peer-reviewed journals. Due to its interdisciplinary nature, this research is of interest for clinicians, researchers and parents.

## 4. Conclusions

Knowledge of the early risk factors related to childhood obesity offers us novel approaches to design prevention strategies. These effective strategies must cover the gestation period, and the first and second year of postnatal life because of the fact that they are considered critical periods for the childhood obesity prevention. Moreover, as many early risk factors as possible, accessibility, cost effectiveness, and sustainability must be taken into account.

The article presented is a novel protocol. The intervention will be integrated into routine primary care health programs and will not require additional effort from professionals or equipment. It could be a new intervention capable of reducing the prevalence of childhood obesity.

## Supplementary Materials

The following are available online at https://www.mdpi.com/1660-4601/17/7/2239/s1.
