# Supplementary material for: Effectiveness of a Multifactorial Intervention in the First 1000 Days of Life to Prevent Obesity and Overweight in Childhood: Study Protocol
Source: Int J Environ Res Public Health. 2020 Mar 26;17(7):2239. doi: 10.3390/ijerph17072239 (PMC7177794; doi:10.3390/ijerph17072239)
Supplement: Supplementary file 1 [file ijerph-17-02239-s001.pdf]

Supplementary A: Consent of the intervention group

CONSENTIMIENTO INFORMADO POR ESCRITO

Título del estudio: Evaluación de la efectividad de una intervención para la prevención de la obesidad infantil, desarrollada en atención primaria durante el embarazo y dos primeros años de vida, sobre la composición corporal a la edad de dos años.
Promotor: Universidad de Cádiz.

Yo (nombre y apellidos) ____________________________________

He leído y comprendido la hoja de información que se me ha entregado.
He podido hacer preguntas sobre el estudio.
He recibido suficiente información sobre el estudio.
He hablado con: ____________________________________ (nombre del investigador).

Comprendo que mi participación es voluntaria.
Comprendo que puedo retirarme del estudio:
1º Cuando quiera
2º Sin tener que dar explicaciones.
3º Sin que esto repercuta en mis cuidados médicos.

Presto libremente mi conformidad para participar en el estudio dentro del grupo de intervención.

FECHA: FIRMA DEL PARTICIPANTE

CONSENTIMIENTO DEL REPRESENTANTE

Título del estudio: Evaluación de la efectividad de una intervención para la prevención de la obesidad infantil, desarrollada en atención primaria durante el embarazo y dos primeros años de vida, sobre la composición corporal a la edad de dos años.
Promotor: Universidad de Cádiz.

Yo (nombre y apellidos) ____ en calidad de ______________ (relación con el participante) de __________________________________ (nombre del participante en el estudio)

He leído y comprendido la hoja de información sobre el estudio.
He podido hacer preguntas sobre el estudio.
He recibido respuestas satisfactorias a mis preguntas
He recibido suficiente información sobre el estudio.
He hablado con: ______________________________ (nombre del investigador)

Comprendo que la participación es voluntaria.
Comprende que puede retirarse del estudio:
1º Cuando quiera
2º Sin tener que dar explicaciones.
3º Sin que esto repercuta en sus cuidados médicos.

Y presto mi conformidad con que __________________________________ (nombre del participante en el estudio) participe en este estudio dentro del grupo de intervención.

FECHA: FIRMA DEL REPRESENTANTE

Supplementary B: Consent of the control group

CONSENTIMIENTO INFORMADO POR ESCRITO

Título del estudio: Evaluación de la efectividad de una intervención para la prevención de la obesidad infantil, desarrollada en atención primaria durante el embarazo y dos primeros años de vida, sobre la composición corporal a la edad de dos años.
Promotor: Universidad de Cádiz.

Yo (nombre y apellidos) ____________________________________

He leído y comprendido la hoja de información que se me ha entregado.
He podido hacer preguntas sobre el estudio.
He recibido suficiente información sobre el estudio.
He hablado con: ____________________________________ (nombre del investigador).

Comprendo que mi participación es voluntaria.
Comprendo que puedo retirarme del estudio:
1º Cuando quiera
2º Sin tener que dar explicaciones.
3º Sin que esto repercuta en mis cuidados médicos.

Presto libremente mi conformidad para participar en el estudio dentro del grupo control.

FECHA: FIRMA DEL PARTICIPANTE

CONSENTIMIENTO DEL REPRESENTANTE

Título del estudio: Evaluación de la efectividad de una intervención para la prevención de la obesidad infantil, desarrollada en atención primaria durante el embarazo y dos primeros años de vida, sobre la composición corporal a la edad de dos años.
Promotor: Universidad de Cádiz.

Yo (nombre y apellidos) ______________________________ en calidad de ____________ (relación con el participante) de ______________________________ (nombre del participante en el estudio)

He leído y comprendido la hoja de información sobre el estudio.
He podido hacer preguntas sobre el estudio.
He recibido respuestas satisfactorias a mis preguntas
He recibido suficiente información sobre el estudio.
He hablado con: ______________________ (nombre del investigador)

Comprendo que la participación es voluntaria.
Comprende que puede retirarse del estudio:
1º Cuando quiera
2º Sin tener que dar explicaciones.
3º Sin que esto repercuta en sus cuidados médicos.

Y presto mi conformidad con que ______________________ (nombre del participante en el estudio) participe en este estudio dentro del grupo control.

FECHA: FIRMA DEL REPRESENTANTE

Supplementary C: Newsletter

Título del estudio: Evaluación de la efectividad de una intervención para la prevención de la obesidad infantil, desarrollada en atención primaria durante el embarazo y dos primeros años de vida, sobre la composición corporal a la edad de 2 años.
Investigador principal: Mercedes Díaz Rodríguez. Profesora UCA.
Coinvestigador principal: Bernardo C. Ferriz Mas. Pediatra EBAP.
Centro: Consultorio Rio San Pedro. c/ Proa s/n. Bda. Rio San Pedro. UGC Ribera.
Puerto Real. Distrito Bahía Cádiz La Janda. Servicio Andaluz de Salud. Teléfono 956 818213

INTRODUCCIÓN

Se le invita a participar en un estudio que ha sido evaluado y aprobado por el Comité Coordinador de la Ética de la Investigación Biomédica de Andalucía.

Por favor, lea esta hoja informativa con atención.

El Dr. ____ le aclarará las dudas que le puedan surgir.

PARTICIPACIÓN VOLUNTARIA

Su participación en este estudio es voluntaria y usted puede anular su decisión y retirar el consentimiento en cualquier momento sin que por ello se altere su relación con el médico ni se produzca perjuicio en su tratamiento o en la atención que usted pueda necesitar.

DESCRIPCIÓN GENERAL DEL ESTUDIO

El objetivo de este estudio consiste en evaluar la efectividad de una intervención de educación sanitaria durante el embarazo y los dos primeros años de vida del niño para mejorar la composición corporal a los dos años de vida. La duración del estudio es de 3 años. En este estudio participarán 250 pacientes/sujetos, 150 niños/as del Grupo de Intervención y 100 niños/as del Grupo Control. La intervención se basará en la introducción del concepto de programación temprana en las visitas ya establecidas en los programas de Atención al embarazo, parto y puerperio y de Salud Infantil y del Adolescente de Andalucía (Niño Sano) que ya se vienen desarrollando en atención primaria, como eje de una estrategia de prevención primaria de la obesidad infantil. Será realizado por un equipo multidisciplinar formado por las matronas, pediatras y enfermeros/as habituales de su centro de salud.

Usted acudirá a su Centro de Salud para las visitas habituales correspondientes a los programas de control de embarazo, parto y puerperio y del programa de salud infantil. El estudio no implica ninguna toma de muestras biológicas ni exploraciones complementarias. Se realizarán las siguientes pruebas: Registro de variables de crecimiento y nutricionales de los niños en los controles habituales de salud infantil realizados por pediatría y enfermería, y registro de los datos sobre gestación y parto realizado por las matronas en los controles habituales de atención al embarazo, parto y puerperio. Los datos del embarazo y dos primeros años de vida serán recogidos en los controles del año y de los dos años a partir de preguntas directas a los padres y de los registros existentes en el historial clínico en el programa Diraya del SAS. Se compararán los resultados del grupo de intervención con los del grupo control (niños/as que cumplen dos años entre noviembre de 2017 y diciembre de 2018) de la UGC Ribera de la Zona Básica de Puerto Real.

BENEFICIOS Y RIESGOS DERIVADOS DE SU PARTICIPACIÓN EN EL ESTUDIO

Se espera que su participación en este estudio le proporcione los siguientes beneficios: Aprender nuevos conocimientos y su aplicación en la práctica sobre la prevención de la obesidad infantil desde el embarazo y dos primeros años de la vida de su hijo/a. Además, la información que se obtenga servirá para ampliar el conocimiento científico sobre la prevención de la obesidad infantil. Es posible que usted no obtenga ningún beneficio para su salud por participar en este estudio. Su inclusión en el estudio no supone ningún riesgo para su salud ni para la de su hijo/a ya que todas las intervenciones previstas son educativas y de consejo nutricional, con recomendaciones avaladas por los organismos y sociedades científicas competentes.

COMPENSACIÓN ECONÓMICA

Su participación en el estudio no supondrá ningún gasto para usted, ni recibirá ninguna compensación económica por ello.

CONFIDENCIALIDAD

Sus datos serán tratados con la más absoluta confidencialidad según lo dispuesto en la Ley Orgánica 15/1999, de 13 de diciembre de Protección de Datos de Carácter Personal. De acuerdo a lo que establece la legislación mencionada, usted puede ejercer los derechos de acceso, modificación, oposición y cancelación de datos, para lo cual deberá dirigirse al investigador responsable del estudio, Dr Bernardo C. Ferriz Mas. Consultorio Rio San Pedro. Teléfono: 956 81 82 13. Los datos recogidos para el estudio estarán identificados mediante un código y sólo el investigador principal/colaboradores podrán relacionar dichos datos con usted y con su historia clínica. Si se publican los resultados del estudio, sus datos personales no serán publicados y su identidad permanecerá anónima.

FINANCIACIÓN

Este estudio está cofinanciado en un 80% por el fondo europeo de desarrollo regional (FEDER), a través de la fundación para la gestión de la investigación biomédica de Cádiz (FCádiz).

RETIRADA DEL CONSENTIMIENTO

Usted puede retirar su consentimiento en cualquier momento sin tener que dar explicaciones. Si usted no desea participar más en el estudio y usted lo quiere así, todos sus datos identificables serán borrados para evitar la realización de un nuevo análisis. También debe saber que puede ser excluido del estudio si los investigadores del estudio lo consideran oportuno. Usted tiene derecho a estar informado de cualquier proyecto de nuevos análisis de los datos recogidos no previsto en este estudio. El investigador siempre deberá solicitarle un nuevo consentimiento que usted podría rechazar. Antes de firmar, lea detenidamente el documento, haga todas las preguntas que considere oportunas, y si lo desea, consúltelo con todas las personas que considere necesario. En caso de duda debe dirigirse al Dr. Bernardo C. Ferriz Mas (Co-Investigador Principal).

Supplementary D: Ethics Committee approval

JUNTA DE ANDALUCIA

CONSEJERÍA DE SALUD
Dirección General de Investigación y Gestión del Conocimiento
Comité Coordinador de Ética de la Investigación Biomédica en Andalucía

DICTAMEN ÉTICO DEL COMITÉ COORDINADOR DE ÉTICA DE LA INVESTIGACIÓN BIOMÉDICA DE ANDALUCÍA

D. Joaquín Alanís López, Secretario del
Comité Coordinador de Ética de la Investigación Biomédica de Andalucía

CERTIFICA

Que este Comité, ha evaluado en los aspectos éticos, el estudio de investigación perteneciente a la convocatoria de subvenciones para la financiación de la I+D+i biomédica y en ciencias de la salud en la provincia de Cádiz, con titulo: **"Evaluación de la efectividad de una intervención para la prevención de la obesidad infantil, desarrollada en atención primaria durante el embarazo y dos primeros años de vida, sobre la composición corporal a la edad de dos años."** y cuya investigadora principal es DOÑA MERCEDES DÍAZ RODRÍGUEZ.

Y considera que:

Se cumplen los requisitos necesarios de idoneidad del protocolo con relación a los objetivos y se ajusta a los principios éticos aplicables a este tipo de estudios y recogidos en la Declaración de Helsinki de 1964, de la Asociación Médica Mundial, y enmiendas posteriores, y en el Convenio del Consejo de Europa de 1996, relativo a los Derechos Humanos y a la Biomedicina,.

Según la información aportada en el manuscrito el tratamiento de los datos de carácter personal de los participantes se ajusta a lo dispuesto en la Ley Orgánica 15/1999 de 13 de diciembre de protección de datos de carácter personal.

Están justificados los riesgos y molestias previsibles para los participantes. Es adecuado el procedimiento para obtener el consentimiento informado.

Por todo lo anterior:

El *Comité Coordinador de Ética de la Investigación Biomédica de Andalucía*, en su reunión del día 26 de abril de 2018 (Acta 04/2018) tras la evaluación ética del citado estudio acuerda emitir un **DICTAMEN FAVORABLE**

Lo que firmo en Sevilla, a 2 de julio de 2018

Joaquin Alanis López

Avda. de la Innovación, s/n. Edificio Arena 1. Apdo. Correos 17.111. 41080 Sevilla
Teléf. 95 500 63 00. Fax 95 540 72 02

Código Seguro De Verificación:	z8Xq22BdFreCboKMQhFqVQ==	Fecha	02/07/2018
Normativa	Este documento incorpora firma electrónica reconocida de acuerdo a la Ley 59/2003, de 19 de diciembre, de firma electrónica.		
Firmado Por	Joaquin Alanis Lopez		
Url De Verificación	https://ws069.juntadeandalucia.es/verifirma/code/z8Xq22BdFreCboKMQhFqVQ==	Página	1/2
